# Interleukin-15 correlates with cytotoxic immune networks in cervical tuberculous lymphadenitis

**DOI:** 10.3389/fimmu.2026.1831890

**Published:** 2026-06-03

**Authors:** Soumaya Bchiri, Khadija Bahrini, Rosane M. B. Teles, Ameni Ben Alaya, Houssem Eddine Kamel, Julie West, Kimia Rategh, Asma Bouzekri, Eya Bousalem, Rim Ouni, Meriem Fassatoui, Helmi Mardassi, Neira Dekhil, Issam Ben Belghith, Rym Lahiani, Emna Romdhane, Meriem Ben-Ali, Soumaya Rammeh, Asma Ferjani, Mamia Ben-Saleh, Mohamed-Ridha Barbouche, Robert L. Modlin, Chaouki Benabdessalem

**Affiliations:** 1 Laboratory of Transmission Control and Immunobiology of Infections, Institut Pasteur de Tunis, Tunis, Tunisia; 2 University Tunis El Manar, Tunis, Tunisia; 3 Faculty of Sciences of Tunis, Tunis, Tunisia; 4 Division of Dermatology, Department of Medicine, University of California, Los Angeles, Los Angeles, CA, United States; 5 Department of Microbiology, Immunology and Molecular Genetics, David Geffen School of Medicine, University of California, Los Angeles, Los Angeles, CA, United States; 6 Faculty of Medicine of Tunis, Tunis, Tunisia; 7 Department of Pathology, Charles Nicolle Hospital, Tunis, Tunisia; 8 Laboratory of Molecular Microbiology, Vaccinology and Biotechnological Development, Institut Pasteur de Tunis, Tunis, Tunisia; 9 Research Laboratory of Antibiotic Resistance, Charles Nicolle Hospital, Tunis, Tunisia; 10 Ear, Nose and throat (ENT) Department, Charles-Nicolle Hospital, Tunis, Tunisia; 11 Department of Microbiology, Immunology, and Infectious Diseases, College of Medicine and Medical Sciences, Arabian Gulf University, Manama, Bahrain

**Keywords:** cervical lymphadenitis, cytotoxicity, IL-15, immunoregulatory marker, tuberculosis

## Abstract

**Introduction:**

Cervical tuberculous lymphadenitis (CTL) represents a localized manifestation of *Mycobacterium tuberculosis* infection in which immune responses are organized within lymphoid tissue. While cytotoxic lymphocyte responses contribute to antimycobacterial immunity, the cytokine networks coordinating these responses in human lymph node tuberculosis remain incompletely defined.

**Methods:**

We performed integrated immune profiling of patients with CTL (n = 60) and non-tuberculous cervical lymphadenopathy (CNTL; n = 44). Immune−gene expression was quantified in peripheral blood and lymph node mononuclear cells by qPCR. Systems-level analyses including principal component and correlation-network approaches were used to define coordinated immune pathways. Serum IL−15 was measured by ELISA, and tissue localization of IL−15 and IL−15Rα was examined by immunohistochemistry.

**Results:**

CTL was characterized by a structured cytotoxic immune program enriched for granulysin, granzyme B, perforin, IFN-γ, and CCL5. Network analysis identified IL-15 as a highly connected hub within this cytotoxic module in CTL. IL-15 transcripts were significantly elevated in both blood and lymph node compartments (p = 0.0003; p = 0.0007, respectively) and strongly correlated with cytotoxic effector genes. Circulating IL-15 concentrations were higher in CTL than CNTL (p < 0.0001) and increased with GeneXpert-defined bacillary burden (AUC 0.73). Immunohistochemistry demonstrated IL-15 and IL-15Rα expression within CD68^+^ macrophages localized to granulomatous regions, consistent with macrophage-mediated IL-15 trans-presentation within sites of infection.

**Conclusions:**

These findings identify IL−15 as a potential central organizer of cytotoxic immune pathways in CTL and highlight IL−15–linked immune signatures as biologically informative features of CTL immunopathogenesis.

## Introduction

According to the World Health Organization (WHO) Global Tuberculosis Report 2024, approximately 8.2 million new and relapse tuberculosis (TB) cases were notified globally. Among these, 84% were pulmonary TB (PTB) and 16% extrapulmonary TB (EPTB). Over recent decades, the incidence of EPTB has increased even in developed countries, while PTB rates have declined. Despite this trend, EPTB continues to receive less attention in TB control programs, possibly due to the perception that it is less transmissible (1).

Tunisia exhibits an epidemiological pattern distinct from the global predominance of pulmonary TB, with extrapulmonary disease accounting for 64.6% of newly diagnosed cases (2). Cervical tuberculous lymphadenitis (CTL) has increased markedly, from 2.3 per 100–000 inhabitants in 1993 to 18 per 100–000 in 2017 (3). Although this distribution is unusually pronounced, multiple studies have documented an increasing relative contribution of extrapulmonary TB, including lymph node involvement, in regions where pulmonary TB incidence has declined (1, 4, 5), positioning Tunisia as a sentinel population for studying CTL immunobiology and biomarker development.

TB lymphadenitis, the most common form of EPTB, typically affects cervical lymph nodes and presents as painless, progressive swelling that may evolve into abscesses or fistulae (6). Differentiating CTL from non-tuberculous lymphadenopathy (CNTL) is clinically critical, as management strategies differ substantially. However, diagnosis remains challenging due to the paucibacillary nature of nodal TB and its overlapping presentation with fungal, bacterial, including atypical mycobacterial, lymphoma and reactive lymphadenopathies (7). Indeed, a small proportion of tuberculous lymphadenitis cases are attributable to non-tuberculous mycobacteria (NTM), accounting for approximately 5–10% of cases, whereas the majority are caused by *Mycobacterium tuberculosis* complex (8). The GeneXpert MTB/RIF assay applied to fine-needle aspirates or biopsy tissue provides rapid bacteriologic confirmation and semi-quantitative bacillary load estimation (9). Tuberculous lymphadenitis generally has a favorable prognosis when diagnosed early and treated appropriately, but delays in diagnosis are common and can adversely affect outcomes (10). However, its management remains challenging due to nonspecific clinical presentation and significant delays in diagnosis and treatment initiation (11), highlighting the need for reliable, non-invasive biomarkers to improve early detection and patient management.

CTL pathogenesis is driven by coordinated interactions between macrophages, T lymphocytes, and cytokine networks that together determine granuloma structure, cytotoxic effector function, and containment of *Mycobacterium tuberculosis (M. tuberculosis)*. Understanding these immune programs, both locally within lymph nodes and systemically in peripheral blood, is essential for defining mechanisms of disease control and failure. Rather than focusing on individual immune mediators in isolation, a systems-level view of immune organization offers the opportunity to identify coherent immune signatures that reflect underlying pathophysiology.

Previous studies of CTL have largely emphasized diagnostic biomarker discovery or antigen-specific interferon-γ release assays, with variable performance in extrapulmonary disease. Understanding these local versus systemic immune mechanisms can reveal biomarkers and inform immunodiagnostic strategies, addressing the limitations of conventional microbiological tests. Current standard blood-based immunodiagnostics such as interferon-γ release assays (IGRAs, e.g., QuantiFERON) demonstrate variable and often reduced diagnostic accuracy for extrapulmonary TB, including lymph node disease (12). We previously demonstrated that the HBHA-based IGRA distinguishes CTL from CNTL with high accuracy, which is further enhanced by incorporating cytotoxic mediators such as granzyme B (13). These findings support the value of targeted profiling of local and systemic immune responses in CTL to identify biomarker panels with improved sensitivity and specificity over current IGRA-based approaches. Accordingly, we selected a gene panel defined based on key immune pathways implicated in cervical TBL pathogenesis, particularly cytotoxic, Th1, and regulatory responses, as supported by our prior work (13). Compared with broader approaches, this targeted qPCR panel enables more sensitive, robust, and quantitative assessment of these biologically relevant pathways across a relatively large number of clinical samples. Moreover, unlike antigen-induced markers used in IGRA assays, which can vary substantially with prior M. tuberculosis exposure, baseline (unstimulated) biomarkers exhibit lower variability and greater reproducibility across populations and disease stages (14–16). While such approaches have clinical utility, they provide limited insight into the immune architecture underlying lymph node tuberculosis. An emphasis on immune pathway organization, rather than single-analyte biomarkers, may better capture the biology of CTL and inform both mechanistic understanding and rational biomarker development.

In this study, we profiled a broad panel of Th1, Th17, regulatory, and cytotoxic immune genes in lymph node and peripheral blood compartments and applied systems-level analyses to define coordinated immune programs underlying CTL. Rather than focusing *a priori* on individual cytokines, we used principal component and correlation-network approaches to identify immune pathways that distinguish tuberculous from non-tuberculous lymphadenopathy. Across these analyses, IL-15 emerged as a highly connected node within a coordinated cytotoxic immune module, prompting further mechanistic investigation.

IL-15 is a macrophage-derived cytokine with a unique mode of action mediated by IL-15Rα–dependent trans-presentation, enabling direct crosstalk between myeloid cells and cytotoxic lymphocytes. Through this mechanism, IL-15 supports the survival, proliferation, and effector differentiation of CD8^+^ T cells and natural killer cells, including induction of cytotoxic granule components and IFN-γ production. Although IL-15 has been implicated in protective immunity and granulomatous responses in tuberculosis, its role as an organizing element within coordinated immune networks in human tuberculous lymph nodes has not been defined (17).

Guided by its central position in our network analyses, we therefore examined IL-15 expression across transcriptomic, serologic, and histologic compartments, including spatial localization of IL-15 and IL-15Rα within lymph node tissue. Our objective was to determine whether IL-15 reflects an underlying cytotoxic immune program operative in CTL and to define its immunological relevance within disease pathogenesis, with potential implications for mechanism-informed biomarker development.

## Materials and methods

### Study design and setting

This case–control study was conducted at Charles Nicolle Hospital in Tunis. Patients presenting with suspected CTL and undergoing lymph node excision biopsy for diagnostic evaluation were prospectively assessed. Of 153 patients initially screened, 104 met the inclusion criteria based on bacteriological, histological, and molecular findings and were included in the analysis.

### Study population

Participants were classified into two groups: confirmed CTL cases (n = 60) and a comparison group with non-tuberculous cervical lymphadenopathy (CNTL, n = 44). CTL was defined by histopathological evidence of epithelioid granulomas and/or necrosis and detection of *M. tuberculosis* DNA in lymph node specimens using the GeneXpert MTB/RIF assay. Semi-quantitative bacillary load results (trace, very low, low, or medium/high) were recorded for CTL patients. Patients receiving anti-tuberculosis treatment prior to sample collection were excluded from the study. CNTL samples were evaluated by GeneXpert MTB/RIF assay and culture to exclude *M. tuberculosis* and non-tuberculous mycobacterial infections.

The CNTL group comprised patients with cervical lymph node enlargement (>1 cm) and alternative diagnoses established by histopathology, including 9 cases of Hodgkin,9 cases of non-Hodgkin lymphoma, 3 cases of carcinoma, and 23 cases of reactive lymphadenopathy. Tuberculosis and non-tuberculous mycobacterial infections were excluded by culture and GeneXpert testing. Pregnant women and individuals with HIV infection, diabetes mellitus, autoimmune diseases, or receiving immunosuppressive therapy were excluded. Written informed consent was obtained from all participants, and the study was approved by the Pasteur Institute of Tunis Ethics Committee (Reference: 2023/02/I/V1).

### Sample collection and processing

Peripheral blood was collected in heparinized tubes, and cervical lymph node tissue was obtained by excisional biopsy. Peripheral blood mononuclear cells (PBMCs) were isolated by Ficoll–Hypaque (Eurobio, Les Ulis, France) density gradient centrifugation and Lymph node mononuclear cells (LNMCs) were prepared by mechanical disruption of tissue using the BD Medimachine system, followed by filtration and density gradient separation, similar to PBMC isolation.

A portion of each lymph node specimen was fixed in formalin and embedded in paraffin for histopathology and immunohistochemistry (IHC). The serum was separated from whole blood and stored at –80 °C until cytokine assays were performed.

### Quantitative PCR gene expression analysis

The mRNA expression of immune-related genes was quantified in PBMCs and LNMCs from CTL and CNTL patients. Total RNA was extracted from cell pellets using the RNeasy Mini Kit (Qiagen, Hilden, Germany), and cDNA was synthesized from 1 µg RNA using random hexamer priming of High-Capacity cDNA Reverse Transcription Kit (Applied Biosystems, Foster City, CA, USA). Quantitative PCR was performed on a 7500 Fast Real-Time PCR System (Applied Biosystems, Foster City, CA, USA) using SYBR Green chemistry.

Primer sequences (**Supplementary Data Sheet 1**) were validated for the following targets: IL-15, Granulysin (GNLY), perforin (PRF1), CCL5 (RANTES), IL10, granzyme B (GZMB), TNF, IFNG, IL1B, IL12A (IL-12p35), EBI3 (IL-27β), IL17A, TGFB1, FOXP3, and the reference gene GAPDH. Reaction specificity was confirmed by melt-curve analysis. Relative gene expression was calculated by the 2-^ΔCt method normalized to GAPDH. Expression levels were compared between CTL and CNTL groups in both PBMC and LNMC compartments.

### Immune marker principal component and network analysis

Principal component analysis (PCA) was performed to explore patterns of immune marker expression in blood and biopsy samples and to assess the separation between CTL and CNTL groups. Prior to analysis, data were log-transformed (if required) and scaled to unit variance (z-score normalization). PCA was conducted in R using the FactoMineR package, and visualizations were generated with factoextra and plotly. Individuals were projected onto the first three principal components (PC1, PC2, and PC3), which capture the highest proportion of variance. Three-dimensional (3D) PCA plots were used to visualize sample distribution, and group dispersion was represented using ellipsoids corresponding to multivariate confidence regions. Two-dimensional projections and variable plots (correlation circles) were used to examine the contribution of each immune marker to the principal components. Variable contributions were visualized based on vector length and color gradient, with higher contributions indicating a stronger influence on the corresponding principal component.

To summarize network connectivity across markers, node strength was defined as the number of significant correlations per marker and visualized using a heatmap (mosaic plot). Together, PCA and correlation-based network analyses were used to compare global immune structure and connectivity patterns between CTL and CNTL groups.

### Serum IL-15 enzyme-linked immunosorbent assay

Serum IL-15 concentrations were quantified using a high-sensitivity human IL-15 ELISA kit (R&D Systems, Minneapolis, MN, USA) according to the manufacturer’s protocol. Briefly, serum samples were diluted 1:2 and incubated in IL-15 antibody–coated microplate wells. After washing, a biotinylated secondary antibody and streptavidin–HRP conjugate were added, followed by TMB substrate. Optical density was measured at 450 nm, and IL-15 concentrations were interpolated from a standard curve generated using recombinant IL-15 (pg/mL). All samples were assayed in duplicate. The detection limit was approximately 1 pg/mL. Receiver operating characteristic (ROC) curve analysis was performed to evaluate the diagnostic performance of IL-15 in distinguishing CTL from CNTL cases. The area under the curve (AUC), sensitivity, and specificity were calculated. Additional analyses were conducted after exclusion of lymphoma cases to assess the impact of clinical heterogeneity on biomarker performance.

Median IL-15 levels were compared between CTL and CNTL groups. CTL patients were also stratified by GeneXpert semi-quantitative category: G1 (trace), G2 (very low), G3 (low), and G4 (medium bacillary load). IL-15 concentrations were evaluated across these subgroups to assess relationships with bacterial burden.

### Immunohistochemistry

Immunohistochemistry (IHC) was performed on formalin-fixed, paraffin-embedded (FFPE) lymph node tissue sections to localize IL-15, IL-15 receptor α (IL-15Rα), and macrophage markers. Serial sections (4 µm) were cut from paraffin blocks, mounted on positively charged glass slides, and dried to ensure tissue adherence. Sections were deparaffinized in xylene and rehydrated through a graded ethanol series to distilled water.

Heat-induced antigen retrieval was performed in citrate buffer (pH 6.0) at near-boiling temperature for 20–30 minutes, followed by cooling to room temperature. Endogenous peroxidase activity was quenched, and nonspecific binding was blocked by incubation with appropriate normal serum prior to primary antibody application. Sections were then incubated for 1 hour at room temperature with primary antibodies against human IL-15 (rabbit monoclonal antibody, Abcam, Cambridge, UK), IL-15Rα (mouse IgG1, clone M162, Santa Cruz Biotechnology, Dallas, TX, USA), and CD68 (mouse IgG2a, clone KP1, Dako/Agilent, Santa Clara, CA, USA), each diluted at optimized concentrations.

Matched isotype control antibodies (rabbit IgG for IL-15; mouse IgG1 and IgG2a for IL-15Rα and CD68, respectively) were applied to serial sections as negative controls. After washing, sections were incubated with species-appropriate biotinylated secondary antibodies, followed by signal amplification using the VECTASTAIN Elite avidin–biotin peroxidase complex (ABC) system (Vector Laboratories, Burlingame, CA, USA). Chromogenic detection was performed using the AEC peroxidase substrate kit (Vector Laboratories, Burlingame, CA, USA), yielding a red reaction product at sites of antigen localization. Sections were counterstained with hematoxylin, rinsed, and mounted using an aqueous mounting medium.

Stained sections were examined by brightfield light microscopy, and representative images were captured from relevant anatomical regions, including granulomatous areas. Quantitative image analysis was performed using ImageJ (NIH) (Fiji (ImageJ) version 2.9.0 (2022 release): https://imagej.net/software/fiji/downloads) with the ImmunoRatio plugin to objectively quantify the proportion of IL-15^+^ and IL-15Rα^+^ staining relative to total nuclear area. For each specimen, five randomly selected high-power fields (400×) were analyzed. Results were expressed as the percentage of positive staining per total nucleated cells. Data are presented as mean ± SD for each group (n = 3 CTL, n = 3 CNTL).

### Statistical analysis

Differences between CTL and CNTL groups were assessed using unpaired two-tailed Student’s *t*-tests for normally distributed variables (after log-transforming skewed qPCR data) or Mann– Whitney *U* tests if distributions were non-normal. Paired comparisons (such as IL-15 expression in blood vs. lymph node within the same patients) were evaluated with Wilcoxon signed-rank test. For multiple group comparisons of IL-15 across bacillary load categories, we used the Kruskal–Wallis test with *post-hoc* Dunn’s test. Pairwise correlations between immune markers were calculated within each group and compartment using Spearman’s rank correlation applied to normalized expression values. To account for multiple comparisons, p-values were adjusted using the Benjamini–Hochberg false discovery rate (FDR) procedure. Correlations were considered statistically significant at an adjusted p-value (FDR) < 0.05 (two-tailed).

Significant correlations were summarized in correlation matrices and visualized as network graphs using Circos plots. In these networks, immune markers were represented as nodes, with node size proportional to node degree (i.e., the number of significant correlations), and edges represented positive correlations, with line thickness proportional to the correlation coefficient (ρ).

To summarize network connectivity across markers, node strength was defined as the number of significant correlations per marker and visualized using a heatmap (mosaic plot).

Correlations were measured by Spearman’s rank test. *P* < 0.05 was considered statistically significant. Analyses were performed using GraphPad Prism 9 and R version 4.5.3 (2026-03–11 ucrt).

## Results

### Patient characteristics

A total of 104 patients were enrolled, including 60 with cervical tuberculous lymphadenitis (CTL) and 44 with non-tuberculous lymphadenopathy (CNTL). The median age was 39 years (range, 18–77) in the CTL group and 38 years (range, 21–71) in the CNTL group. The CTL group showed a female predominance, whereas sex distribution was balanced in the CNTL group. All CTL patients had microbiologically confirmed *M. tuberculosis* infection in lymph node tissue by GeneXpert MTB/RIF assay. GeneXpert semi-quantitative results among CTL cases were distributed as follows: 33% trace, 33% very low, 23% low, and 10% medium, reflecting a range of bacillary burdens. The CNTL group included patients with reactive lymphadenitis, other bacterial adenitis, lymphoma, and metastatic cancer, all of whom were GeneXpert-negative for *M. tuberculosis*. Baseline clinical features (aside from TB-specific findings) were similar between groups. All participants were presumed to have received BCG vaccination at birth, as it is mandatory in Tunisia, resulting in no variability between TBL and NTBL groups. Demographic and clinical characteristics are summarized in **Table 1**.

**Table 1:** Demographic and clinical characteristics of the study population.

Parameter		CTL n=60	CNTL n=44
Age, Median (range),(Years)		39 (18–77)	38 (21-71)
Sex, Male/Female (n)		15/45	24/20
Smoking (n)		5	6
Alcoholism (n)		1	1
HIV positive (n)		0	0
GeneXpert Results			
Grade of infection	Positive trace	20	–
	Positive very low	20	–
	Positive low	14	–
	Positive (medium/high)	6	–
Negative		0	44

CTL group included patients with confirmed *Mycobacterium tuberculosis* infection based on GeneXpert results, while CNTL group included individuals who tested negative. Age is expressed as median with range. Smoking, alcoholism, and HIV status are listed as the number of affected individuals in each group. GeneXpert results are categorized by semi-quantitative bacterial load as reported by the assay: trace, very low, low, and positive (medium/high). The CNTL group showed no positive GeneXpert results. CTL, tuberculosis-positive control group; CNTL, tuberculosis-negative control group; MTB, *Mycobacterium tuberculosis*; HIV, human immunodeficiency virus; n, number.

### Systems−level immune profiling distinguishes CTL from CNTL

**Figure 1:**
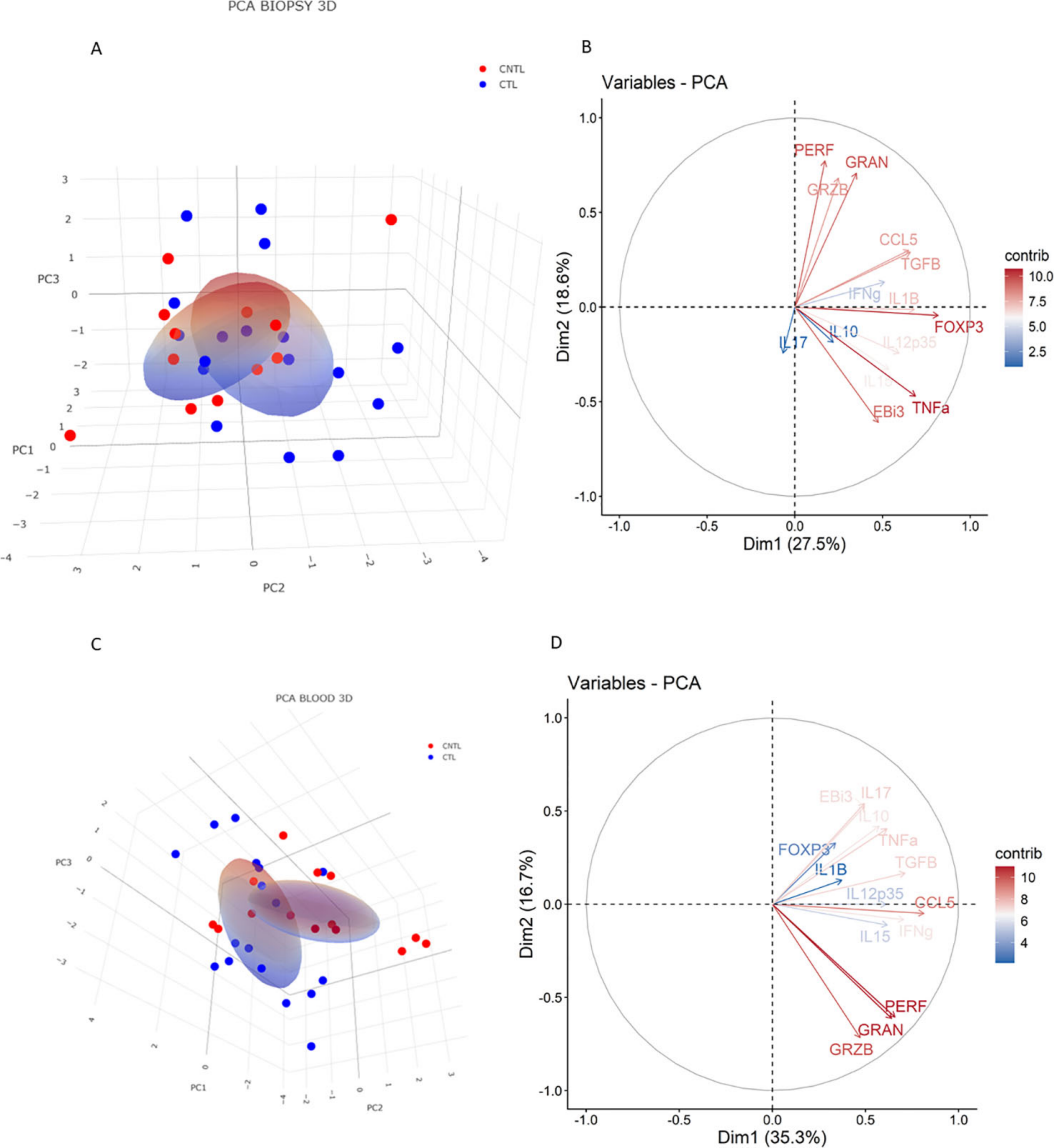
PCA of immune marker expression in blood and biopsy samples. PCA was performed to assess the separation between CTL and CNTL patients based on immune marker expression. **(A, C)** Three-dimensional PCA score plots (PC1–PC3) showing the distribution of samples colored by group (CTL in blue, CNTL in red). Ellipsoids represent group dispersion in 3D space. **(B, D)** PCA variable plots (correlation circles) illustrating the contribution of immune markers to the principal components. Arrow length and direction indicate contribution and correlation, while color intensity reflects contribution level (blue: low, red: high). In blood samples, PC1 accounts for the largest proportion of variance (~35–37%) and is mainly driven by cytotoxic markers (PERF, GRAN, GRZB) and inflammatory cytokines (TNFα, IL-17). In biopsy samples, PC1 explains ~27–30% of the variance and is primarily driven by GRZB, GRAN, PERF, TNFα, and FOXP3.Overall, PCA reveals partial separation between CTL and CNTL groups, driven by coordinated cytotoxic and inflammatory immune responses, with distinct profiles between blood and tissue compartments. PCA, principal component analysis; CTL, cervical tuberculous lymphadenitis; CNTL, non-tuberculous cervical lymphadenopathy; PERF, perforin; GRAN, granulysin; GRZB, granzyme B; IL-10, interleukin 10; TNFα, tumor necrosis factor alpha.

To define immune programs associated with CTL, we applied principal component analysis (PCA) to qPCR−derived immune gene expression data from lymph node and blood compartments. In lymph node mononuclear cells, CTL and CNTL samples showed partial separation along PC1, PC2 and PC3, reflecting modest but consistent differences in immune state (**Figures 1A, B**) (**Supplementary Figure 1**). CTL samples clustering was mainly driven by gene expression of cytotoxic markers, including PRF1, GNLY, GZMB, IFNG, and CCL5. In contrast, CNTL samples exhibited greater dispersion and were influenced by regulatory or inflammatory transcripts such as FOXP3, EBI3, and TNF.

In peripheral blood, PCA revealed a more pronounced separation between groups (**Figure 1C**) (**Supplementary Figure 2**). CTL samples clustered tightly and were driven predominantly by coordinated expression of cytotoxic genes, including GNLY, GZMB, and PRF1. These markers contributed most strongly to the CTL−associated variance, whereas IL17A, TGFB1, and TNF were more closely associated with CNTL samples.

These findings indicate that CTL is characterized by a structured cytotoxic transcriptional program pronounced at both sites of disease.

### IL−15 integrates cytotoxic immune networks in CTL

**Figure 2:**
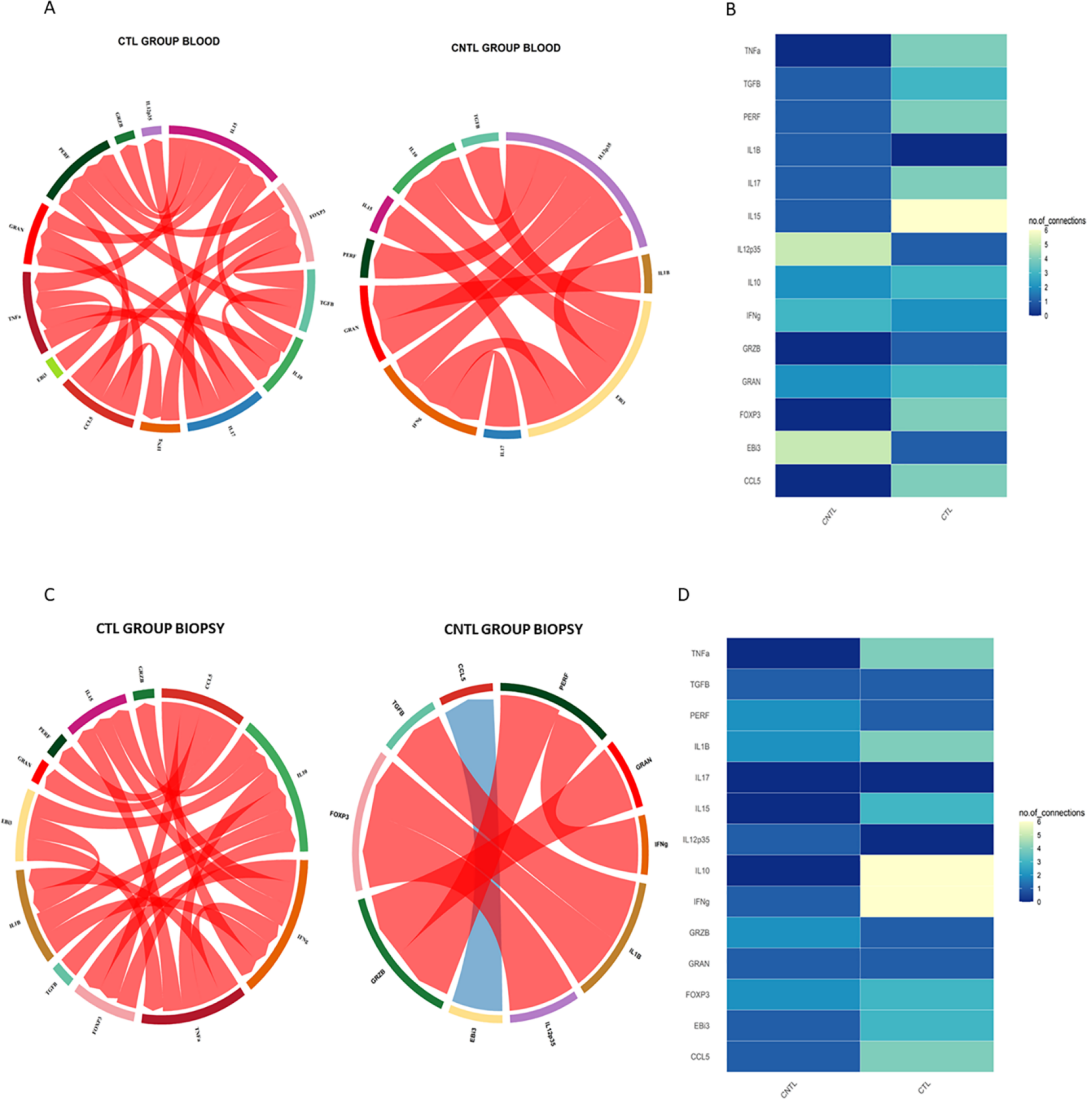
Network analysis of biomarker correlation matrices in CTL and CNTL patients in blood and biopsy. **(A, C)** Spearman correlation matrices of the biomarker expression levels in each study group were built and Circos plots illustrate the correlation networks. Each bar represents a different parameter. The length of each bar is proportional to the number of significant correlations. The connecting lines represent statistically significant correlations (p < 0.05). Red connecting lines represent positive correlations. The thickness of the connecting lines is proportional to the Spearman correlation coefficient value. Markers which did not exhibit statistically significant correlations are shown in the Circos plots. **(B, D)** Node analysis: heatmap shows the number of statistically significant correlations involving each marker per clinical group. CTL, cervical tuberculous lymphadenitis; CNTL, non-tuberculous cervical lymphadenopathy; Spearman correlation, non-parametric measure of rank correlation; Circos plots, circular visualization of network connections; Node, a point or marker within a correlation network; Heatmap, graphical representation of data where values are depicted by color.

To examine immune coordination beyond individual gene effects, we performed correlation−network analysis. CTL samples displayed dense and highly interconnected networks in both blood and lymph node compartments, whereas CNTL samples showed sparse and weak inter−marker correlations (**Figures 2A, C**). Within CTL networks, IL−15 emerged as a central node, exhibiting significant positive correlations with key cytotoxic mediators including GNLY, PRF1, GZMB, IFNG, and CCL5. This pattern was observed systemically and locally, supporting a role for IL−15 in organizing cytotoxic immune architecture rather than acting as an isolated cytokine.

Heatmap−based node−strength analysis confirmed IL−15 among the most highly connected immune markers in CTL blood (**Figure 2B**), while IL−15, IFNG, CCL5 and IL−10 dominated connectivity within CTL lymph nodes (**Figure 2D**). These coordinated relationships were largely absent in CNTL samples, underscoring that *M. tuberculosis* infection induces a synchronized immune network centered on cytotoxic lymphocyte function.

### Differential immune gene expression highlights IL−15 consistency across compartments

**Figure 3:**
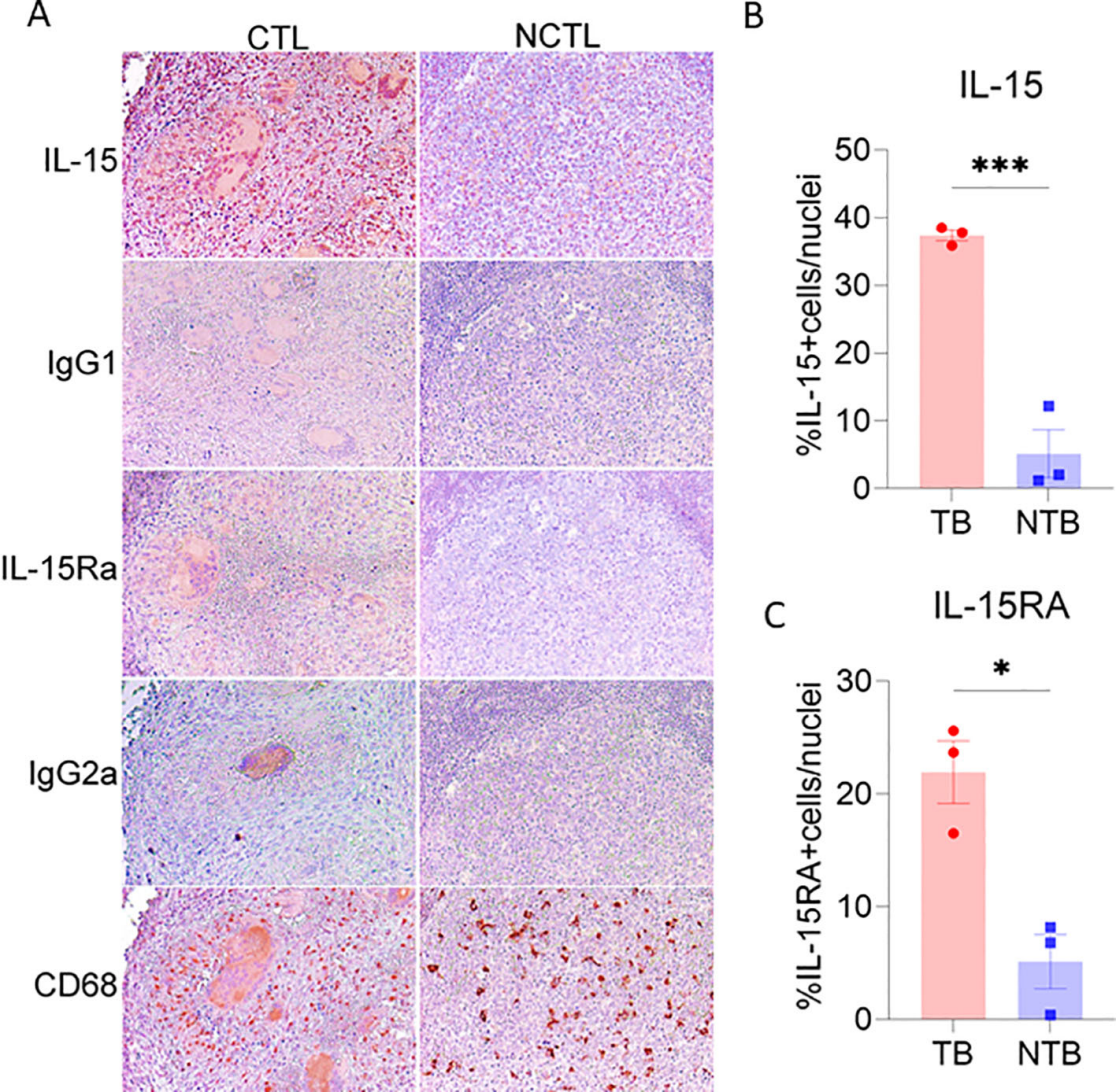
Differential expression of immune-related genes in blood and biopsy from CTL (n = 17) and CNTL (n = 14) patients. Gene expression levels were quantified by qPCR in lymph node mononuclear cells (LNMCs) and PBMCs isolated from CTL and CNTL patients. Red dots represent CTL patients and blue dots represent CNTL individuals. Data are presented as individual values with median ± interquartile range (IQR). The biomarkers include Perforin, IL-15, CCL5, IL-10 and Granulysin. Statistical comparisons were performed using unpaired two-tailed t-tests. Significant differences were observed in the expression of: Perforin (*p = 0.0052*) IL-15 (*p = 0.0007*), IL-10 (*p = 0.0102*), CCL5 (*p = 0.0160*) in biopsy while in blood IL-15 (p = 0.0003) and Granulysin *(p=0.0431)*, A p < 0.05 was considered statistically significant. CTL, cervical tuberculous lymphadenitis; CNTL, non-tuberculous cervical lymphadenopathy; LNMCs, lymph node mononuclear cells; PBMCs, peripheral blood mononuclear cells; qPCR, quantitative polymerase chain reaction; IL, interleukin; CCL5, chemokine (C-C motif) ligand 5. * mean p < 0.05, ** mean p < 0.01, *** mean p ≤ 0.001, **** mean p < 0.0001.

Targeted qPCR analysis revealed distinct expression profiles between CTL and CNTL (**Figure 3**). In lymph nodes, IL-15 (p = 0.0007), IL10 (p = 0.0102), and *CCL5* (p = 0.0160) transcripts were significantly elevated in CTL compared with CNTL. PRF1 expression was paradoxically higher in CNTL samples (p = 0.0052). No significant differences were observed among the other immune biomarkers analyzed (**Supplementary Figure 3**).

In peripheral blood, IL-15 mRNA levels were significantly higher in CTL than in CNTL (p = 0.0003), while GNLY was modestly elevated (p = 0.0431) (**Figure 3**). Other transcripts (*PRF1, CCL5, IL10*) showed no significant systemic differences (all p > 0.1) (**Supplementary Figure 4**). Expression levels were lower overall and more variable in blood than in lymph nodes. Collectively, these results identify IL-15 as one of the most consistently upregulated immune genes in CTL patients, both systemically and at the site of the infection.

### Serum IL−15 reflects cytotoxic immune activation and disease burden

**Figure 4:**
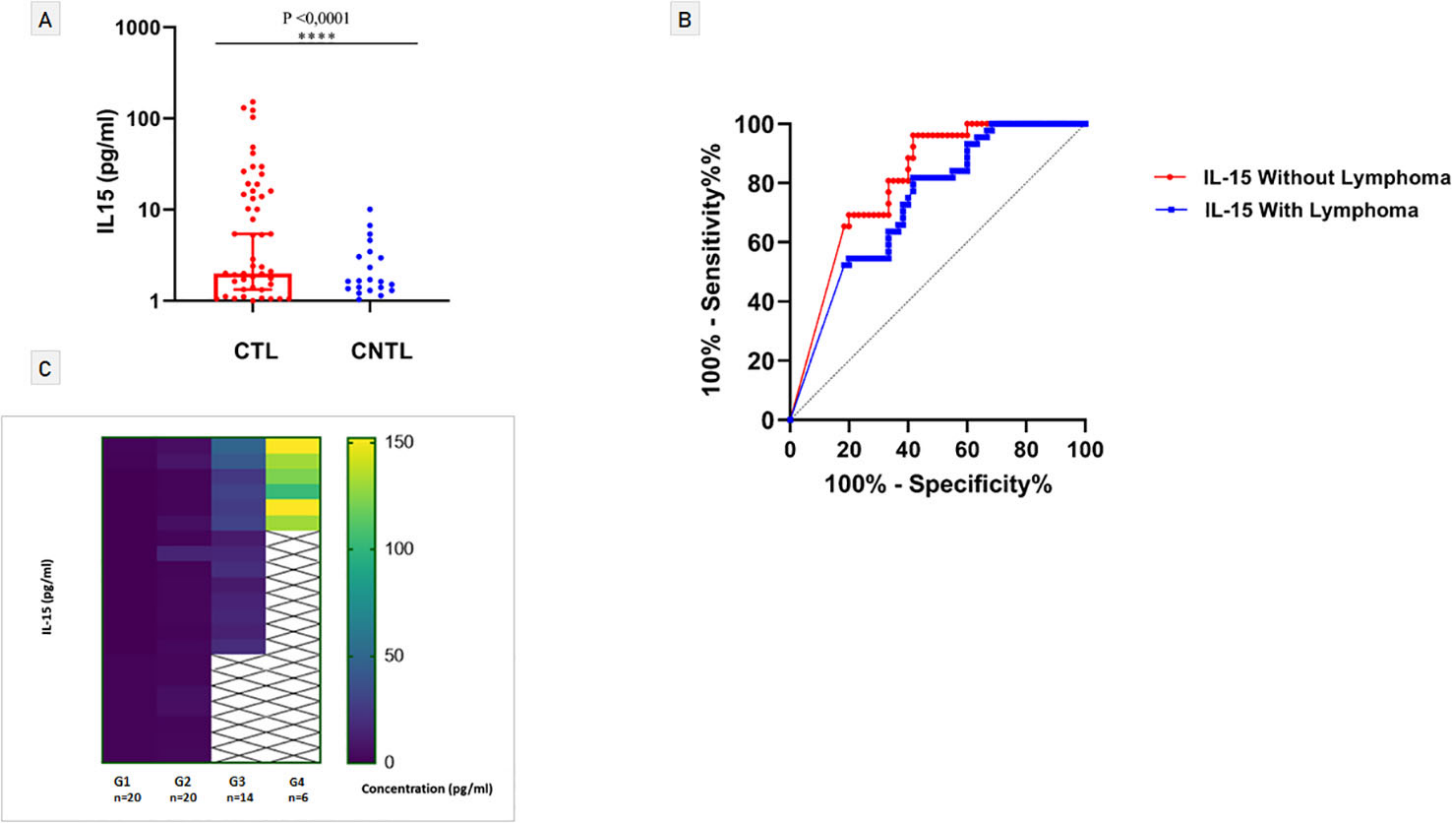
Immunoregulatory and diagnostic relevance of serum IL-15 in cervical tuberculous lymphadenitis (CTL) (A) **(A)** Median serum IL-15 concentrations (pg/mL) in CTL patients (n = 60) compared with non-tuberculous cervical lymphadenopathy controls (CNTL, n = 44), measured by ELISA and presented on a logarithmic scale (median ± IQR). **(B)** ROC curve showing the diagnostic performance of serum IL-15 for CTL (AUC = 0.73, 95% CI: 0.63–0.82; p < 0.0001). After exclusion of lymphoma cases from the CNTL group, the diagnostic performance improved, with an AUC of 0.808 (95% CI: 0.7172–0.8988; p < 0.0001), sensitivity of 69.23% (95% CI: 50.01–83.50%), and specificity of 80.00% (95% CI: 68.22–88.17%). **(C)** Heatmap of serum IL-15 levels by bacillary load (G1–G4), showing significant differences across groups (Kruskal–Wallis test, p < 0.0001). CTL, cervical tuberculous lymphadenitis; CNTL, non-tuberculous cervical lymphadenopathy; ROC, receiver operating characteristic; AUC, area under the curve; CI, confidence interval; ELISA, enzyme-linked immunosorbent assay. **** mean p<0.0001.

Serum IL-15 concentrations measured by ELISA were significantly higher in CTL patients than in CNTL (**Figure 4A**; p < 0.0001). IL-15 was detectable in most CTL sera (mean = 17 pg/mL, range 5–50 pg/mL) but remained near or below detection limits in CNTL (<5 pg/mL). ROC curve analysis including all CNTL cases, notably lymphoma patients, showed moderate discrimination (AUC = 0.73; 95% CI, 0.63–0.82; p < 0.0001) (**Figure 4B**). However, sensitivity analysis excluding lymphoma cases improved the diagnostic performance of IL-15 (AUC = 0.80), along with increased sensitivity (69%) while maintaining comparable specificity (80%), highlighting its enhanced utility in a more clinically homogeneous non-TBL group.

Interestingly, when CTL patients were stratified by GeneXpert bacillary load (trace to medium), IL-15 concentrations increased progressively with bacterial burden (**Figure 4C**; p < 0.0001 by Kruskal–Wallis test), suggesting that circulating IL-15 may reflect disease activity and/or bacillary load in TB lymphadenitis.

### IL−15 and IL−15Rα localize to macrophage−rich granulomatous regions

**Figure 5:**
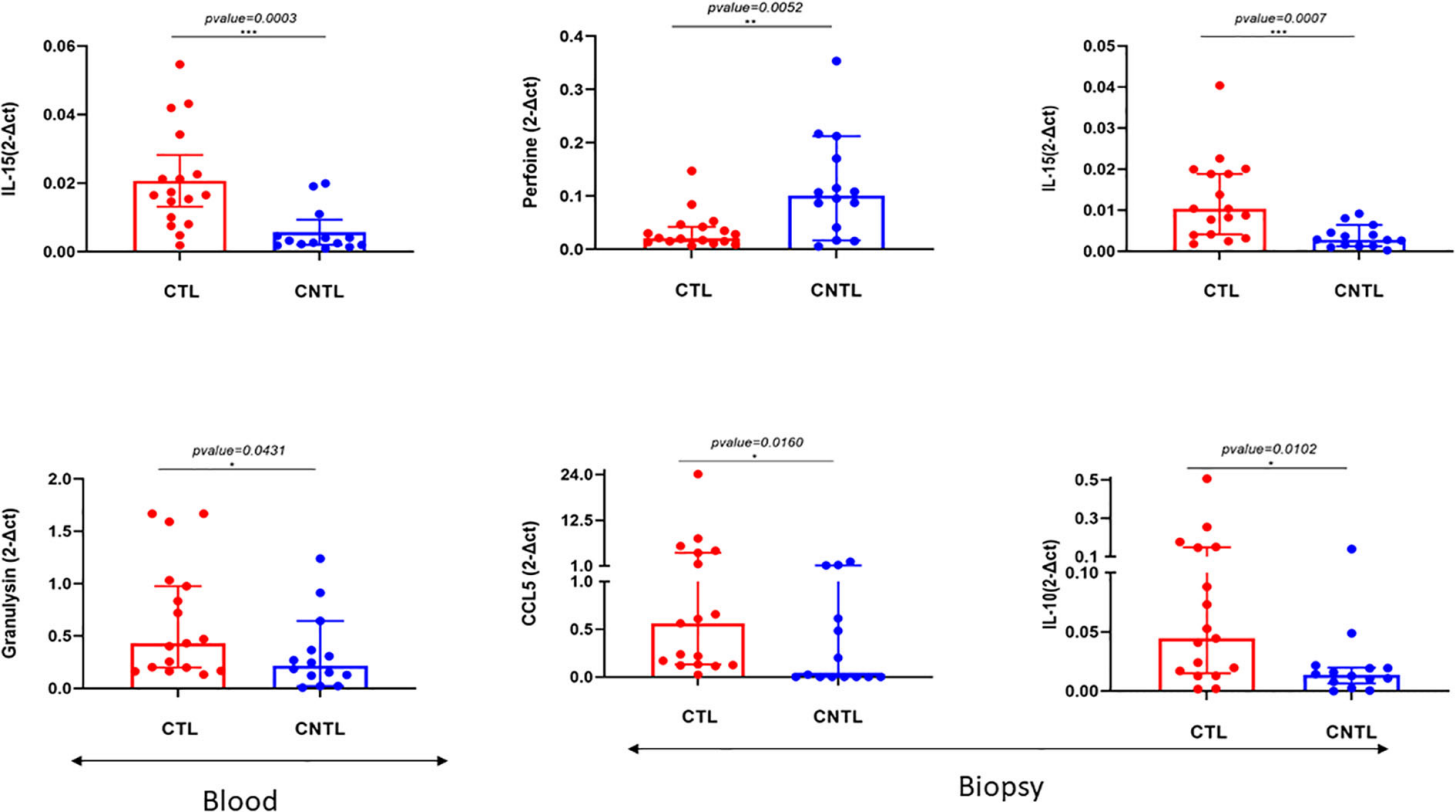
Immunohistochemical analysis of IL-15 and associated markers in cervical lymphadenitis tissues **(A)** Representative immunohistochemical staining of cervical lymph node sections from patients with CTL and CNTL showing IL-15, IL-15Rα, and CD68 expression. IgG1 and IgG2a were used as isotype controls. CTL samples exhibited increased IL-15 and IL-15Rα expression accompanied by enhanced CD68^+^ macrophage infiltration compared with CNTL. Scale bars and magnifications are consistent across panels. Representative data from one of three patients are shown. **(B)** Quantification of IL-15^+^ and **(C)** IL-15Rα^+^ cells expressed as the percentage of positive cells per total nuclei in tissue sections from CTL and CNTL groups. Data are shown as mean ± SD (n = 3 per group); points represent individual biological replicates. Statistical significance was assessed using an unpaired two-tailed Student’s *t*-test. CTL, cervical tuberculous lymphadenitis; CNTL, cervical non-tuberculous lymphadenitis; IL-15, interleukin-15; IL-15Rα, interleukin-15 receptor alpha; IHC, immunohistochemistry. * mean p< 0.05; ** mean p<0.01; *** mean p≤ 0.001.

Immunohistochemical analysis revealed strong expression of IL−15 and IL−15Rα within CD68^+^ macrophages located in granulomatous regions of CTL lymph nodes (**Figure 5**) (**Supplementary Figure 5**). Staining was concentrated in epithelioid macrophages surrounding areas of necrosis, consistent with sites of active immune–pathogen interaction. Staining was predominantly cytoplasmic and localized to epithelioid macrophages and surrounding mononuclear cells at the periphery of caseous necrosis. IL-15Rα displayed a similar spatial distribution, supporting co-expression within macrophage populations. In contrast, CNTL tissues showed minimal IL−15 or IL−15Rα staining and lacked organized granulomatous architecture.

Quantitative analysis using the ImageJ ImmunoRatio plugin demonstrated a significant enrichment of IL-15^+^ cells in CTL samples (37.4% ± 1.35% of total nuclei) compared with CNTL samples (5.13% ± 6.13%; p < 0.001). Likewise, the proportion of IL-15Rα^+^ cells was substantially higher in CTL granulomas (21.93% ± 4.80%) than in CNTL tissues (5.13% ± 4.16%; p < 0.001). Together, these findings suggest that IL-15 and IL-15Rα are co-expressed within macrophage-rich CTL granulomas, supporting that IL-15 is tightly integrated within a cytotoxic immune transcriptional program.

## Discussion

Cervical tuberculous lymphadenitis (CTL) represents a localized manifestation of *M. tuberculosis* infection in which immune responses are organized within lymphoid tissue rather than the lung parenchyma. While extrapulmonary tuberculosis accounts for a substantial fraction of global TB cases, the immunological architecture underlying lymph node disease remains less well defined than that of pulmonary TB. In this study, we applied a systems-level immunologic approach to CTL, integrating transcriptional profiling, immune-network analysis, serologic measurements, and spatial localization to define coordinated immune programs operative in human lymph nodes tuberculosis.

Rather than identifying isolated immune markers, our analyses revealed that CTL is characterized by a highly structured cytotoxic immune program, most prominently expressed within lymph node tissue. Principal component analysis revealed that IL−15 clustered tightly with cytotoxic molecules including GNLY, GZMB, PRF1, and CCL5 indicating coordinated activation of cytotoxic T−cell and NK−cell pathways. This program was consistently observed in both peripheral blood and at the site of infection, indicating that the IL-15–associated immune signature is shared across compartments, while remaining more coherent at the tissue level, underscoring the importance of local immune organization in CTL pathogenesis. This aligns with IL−15’s established biological functions, including promoting survival, proliferation, and activation of NK cells and CD8^+^ T cells and enhancing IFN−γ production during mycobacterial infection (18, 19). Prior studies in both humans and non−human primates similarly highlight IL−15 as a driver of cytotoxic effector expansion within granulomatous inflammation (20). Our findings extend these observations to lymph node TB, demonstrating that IL−15 emerges as a central cytotoxic module that differentiates CTL from other lymph node pathologies. Indeed, correlation-network analysis further supported a central role for IL-15 in CTL immunobiology. CTL patients exhibited a densely interconnected cytokine effector network, with IL-15 strongly correlated with cytotoxic mediators, whereas CNTL samples showed sparse and poorly coordinated interactions. This contrast suggests that active *M. tuberculosis* infection within lymph nodes induces a structured immune architecture organized around IL-15. Similar cytokine hub–driven network configurations have been reported in TB meningitis and pulmonary TB, where dominant inflammatory nodes shape the broader immune milieu (20), reinforcing the mechanistic relevance of IL-15 in TB pathogenesis.

Serum IL-15 levels mirrored these tissue-level findings, showing significant elevation in CTL. Our findings support IL-15 as a promising biomarker for TBL, with a performance profile suggesting higher specificity than sensitivity, making it more suitable as a rule-in rather than a standalone diagnostic tool. Its diagnostic accuracy improved in more clinically homogeneous groups, highlighting its potential utility as an adjunctive biomarker alongside existing tests. However, its moderate sensitivity and variability across patient subgroups underscore the need for validation in larger cohorts and in combination with other biomarkers to enhance overall diagnostic performance. Interestingly, when CTL patients were stratified by GeneXpert bacillary load, IL-15 concentrations increased progressively with bacterial burden suggesting that circulating IL-15 may reflect disease activity and/or bacillary load in TB lymphadenitis.

Importantly, a similar association between IL-15 and mycobacterial burden was reported by Heslop et al. using 16S rRNA quantification (21), whereas Kumar et al. observed no such relationship in pulmonary TB when relying on smear microscopy (22). These discrepancies likely reflect differences in methodological sensitivity, as smear grading provides a crude estimate of bacillary load compared with molecular approaches, particularly in paucibacillary disease such as lymph node TB. These observations emphasize that biomarker relevance is best understood in the context of immune pathway organization, rather than as isolated measurements divorced from mechanism.

The biological plausibility of this role is reinforced by the known immunology of IL-15. Unlike many cytokines that act through soluble signaling, IL-15 signals primarily via IL-15Rα–mediated trans-presentation, enabling macrophages and other myeloid cells to directly activate neighboring CD8^+^ T cells and natural killer cells. Through this mechanism, IL-15 promotes cytotoxic lymphocyte survival, granule loading, and IFN-γ production features that align precisely with the transcriptional programs observed in CTL lymph nodes. Our immunohistochemical findings extend this framework by demonstrating co-localization of IL-15 and IL-15Rα within CD68^+^ macrophages in granulomatous regions, providing spatial evidence for macrophage-anchored IL-15 signaling in human lymph node tuberculosis.

Together, these data support that macrophages in CTL granulomas are a prominent source of IL-15 and IL-15Rα expression and are embedded within regions enriched for cytotoxic immune programs, consistent with a role for IL-15 in structuring local immune responses. By trans-presenting IL-15, macrophages may sustain localized cytotoxic lymphocyte activation within granulomas, contributing to containment of *M. tuberculosis* in lymphoid tissue. This model is consistent with prior observations in pulmonary TB and non-human primate models, but extends those findings by defining IL-15 centered immune organization specifically within human lymph node disease (23).

Although our findings identify IL-15 as a potential central hub within cytotoxic immune networks in CTL, the present study does not directly test whether IL-15 is required to drive this program. Functional perturbation experiments, such as blockade of IL-15 signaling or exogenous IL-15 stimulation of lymph node–derived immune cells, will be important in future studies to determine whether IL-15 is mechanistically necessary for cytotoxic lymphocyte activation in lymph node tuberculosis. Nevertheless, the consistent association of IL-15 with multiple cytotoxic effector pathways across transcriptional, serologic, and spatial analyses strongly supports its role as an integrative component of CTL immune organization. An important direction for future studies would be the direct phenotypic characterization of cytotoxic CD8^+^ T cells and NK cells, along with assessment of the coordinated expression of cytotoxic effector genes (GNLY, GZMB, PRF1, IFNG), to further support the presence of cytotoxic immune activity within CTL granulomas.

This study has other limitations. The cohort size limited detailed subgroup analyses and precluded multivariable modeling to assess interactions among immune markers. Additionally, the CNTL comparison group encompassed heterogeneous non-tuberculous etiologies, although the absence of coordinated cytotoxic networks across all control subtypes strengthens the specificity of our findings. Future studies incorporating longitudinal sampling and expanded immune profiling will be important to define how IL-15 centered networks evolve with treatment and resolution of disease.

In summary, our findings identify CTL as a disease state defined by coordinated cytotoxic immune organization within lymph nodes, with IL-15 emerging as a key integrator linking macrophage activation to cytotoxic lymphocyte effector function. By prioritizing immune pathways and network architecture over individual analytes, this work provides a mechanistic framework for understanding lymph node tuberculosis and highlights IL-15 associated immune programs as biologically informative features of CTL pathogenesis, with potential implications for mechanism-guided biomarker development.

## Data Availability

All datasets related for this study are included in the manuscript and/or the **Supplementary Material**.
