# Supplementary material for: Interleukin-15 correlates with cytotoxic immune networks in cervical tuberculous lymphadenitis
Source: Front Immunol. 2026 Jun 3;17:1831890. doi: 10.3389/fimmu.2026.1831890 (PMC13271968; doi:10.3389/fimmu.2026.1831890)
Supplement: Supplementary file 1 [file DataSheet1.docx]

Supplementary Table 1: Sequences of primers used for qPCR

| **Gene** | **5'-3' primer sequence** | **Tm (°C)** | **amplicon size (pb)** |
| --- | --- | --- | --- |
| **granzyme B (sens)** | TAGCAACAAGGCCCAGGTGA | 62 | 130 |
| **granzyme B (anti-sens)** | GCACTTTCGATCTTCCTGCAC | 64 |  |
| **granulysine (sens)** | GTGTTTCCAATGCTGCGACC | 62 | 130 |
| **granulysine (anti-sens)** | TGCTGGGCAGTTTCTCCG | 58 |  |
| **perforine (sens)** | ACTCCTAAGCCCACCAGCAA | 62 | 134 |
| **perforine (anti-sens)** | CTGTAGAAGCGGCACTCCAC | 64 |  |
| **IFN-γ (sens)** | TTTGGGTTCTCTTGGCTGTT | 63.5 | 155 |
| **IFN-γ (anti-sens)** | TCCATTATCCGCTACATCTGAA | 63.5 |  |
| **IL-15 (sens)** | ACAGAAGCCAACTGGGTGAA | 62.3 | 160 |
| **IL-15 (anti-sens)** | GCACTTCATTGCTGTTACTTTGC | 61.5 |  |
| **TGF-β (sens)** | GCCCTGGACACCAAC | 60.2 | 120 |
| **TGF-β (anti-sens)** | CTGGTCCAGGCTCCA | 61.3 |  |
| **Foxp3 (sens)** | AGCTGGAGTTCCGCAAGAAAC | 65 | 167 |
| **Foxp3 (anti-sens)** | TGTTCGTCCATCCTCCTTTCC | 65.3 |  |
| **IL-1 β (sens)** | AATCTGTACCTGTCCTGCGTGTT | 62.9 | 144 |
| **IL-1 β (anti-sens)** | TGGGTAATTTTTGGGATCTACACTCT | 63.2 |  |
| **TNF-α (sens)** | CCTGCCCCAATCCCTTTATT | 65.7 | 129 |
| **TNF-α (anti-sens)** | CCCTAAGCCCCCAATTCTCT | 65.2 |  |
| **IL-17 A (sens)** | ACCAATCCCAAAAGGTCCTC | 63.9 | 162 |
| **IL-17 A (anti-sens)** | TGGATGGGGACAGAGTTCAT | 64.4 |  |
| **CCL5 (sens)** | TTTGCCTGTTTCTGCTTGCT | 64.2 | 155 |
| **CCL5 (anti-sens)** | CTGCTGCTGTGTGGTAGAATC | 60.5 |  |
| **IL-10 (sens)** | CGAGATGCCTTCAGCAGAGT | 64 ,7 | 145 |
| **IL-10 (anti-sens)** | CCCTTAAAGTCCTCCAGCAA | 63 |  |
| **IL12α p35 (sens)** | CCACTCCAGACCCAGGAATGT | 63.3 | 134 |
| **IL12α p35 (anti-sens)** | CCTCCACTGTGCTGGTTTTATCT | 62.9 |  |
| **Ebi3 (sens)** | TCATTGCCACGTACAGGCTC | 60.5 | 130 |
| **Ebi3 (anti-sens)** | GGGTCGGGCTTGATGATGTG | 62.5 |  |
| **GAPDH (sens)** | AAGGTGAAGGTCGGAGTCAAC | 64 | 102 |
| **GAPDH (anti-sens)** | GGGGTCATTGATGGCAACAATA | 64 |  |
