# Supplementary material for: Interleukin-15 correlates with cytotoxic immune networks in cervical tuberculous lymphadenitis
Source: Front Immunol. 2026 Jun 3;17:1831890. doi: 10.3389/fimmu.2026.1831890 (PMC13271968; doi:10.3389/fimmu.2026.1831890)
Supplement: Supplementary file 2 [file DataSheet2.docx]

**Supplementary Figures**


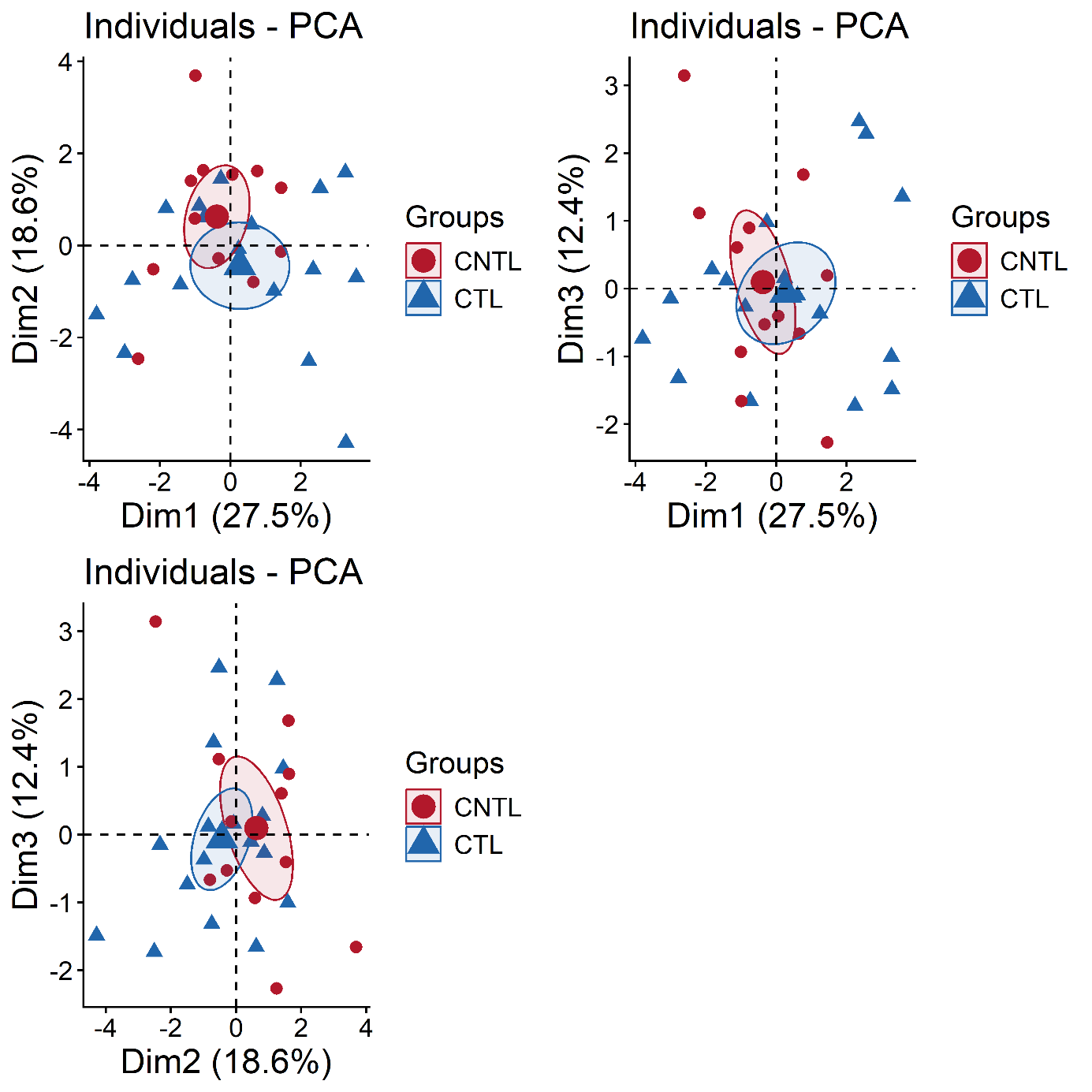


**Supplementary Fig.1: *PCA of immune marker expression in biopsy samples***. PCA was performed to assess the separation between CTL and CNTL patients based on immune marker expression.


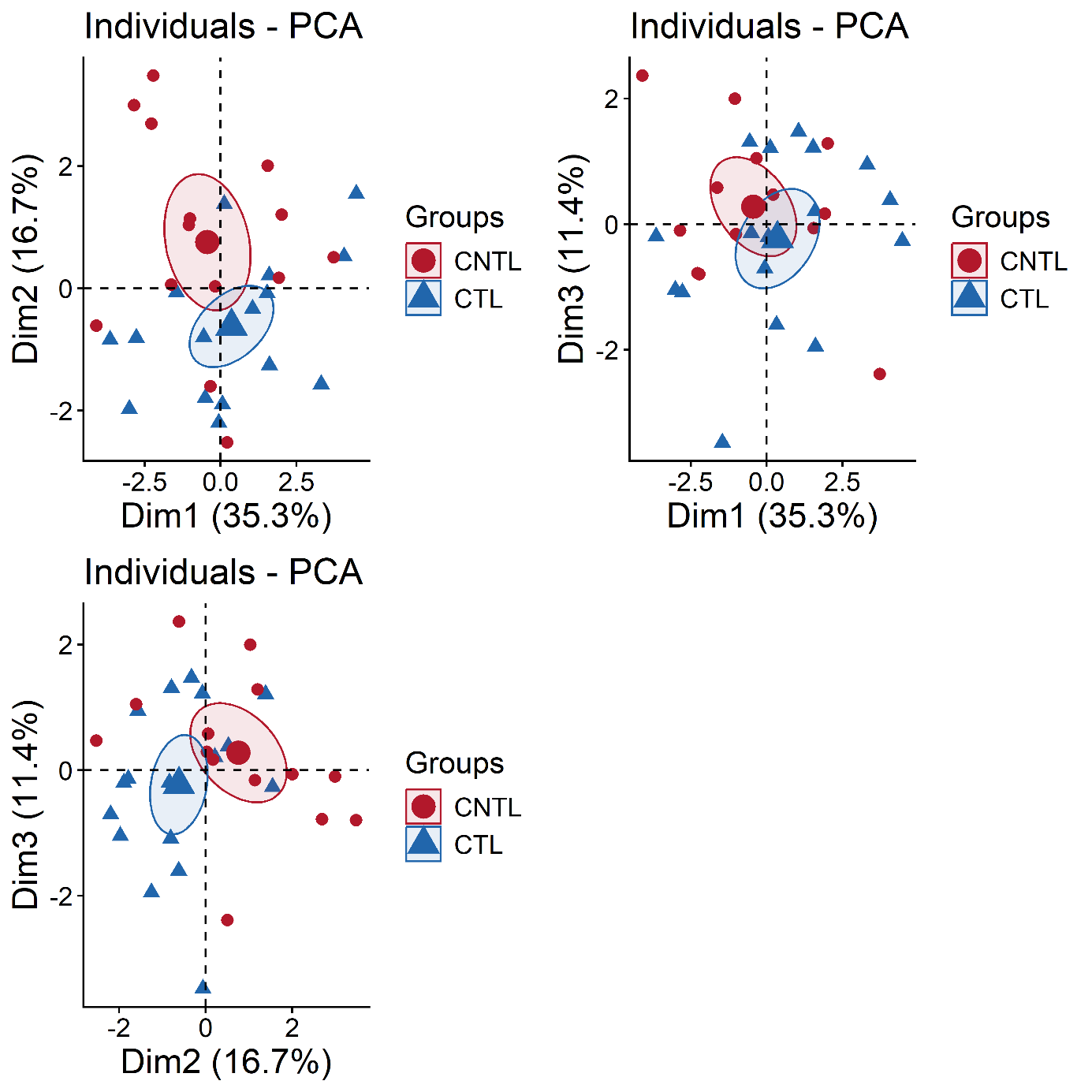


**Supplementary Fig.2: *PCA of immune marker expression in Blood samples***. PCA was performed to assess the separation between CTL and CNTL patients based on immune marker expression.





**Supplementary Fig.3**. ***Differential expression of immune-related genes in lymph node mononuclear cells from CTL (n = 17) and CNTL (n = 14) patients.*** Gene expression levels were quantified by qPCR in lymph node mononuclear cells isolated from CTL and CNTL patients. Red dots represent CTL patients and blue dots represent CNTL individuals. Data are presented as individual values with median ± interquartile range (IQR). The biomarkers include Granzyme B, Granulysin, TNF-α, IL-1β, IFN-γ, FOXP3, IL-12p35, EBI3, IL-17, and TGF-β. Statistical comparisons were performed using unpaired two-tailed t-tests. Non-significant comparisons are labeled as *ns*. A p < 0.05 was considered statistically significant.

*CTL, cervical tuberculous lymphadenitis; CNTL, non-tuberculous cervical lymphadenopathy; LNMCs, lymph node mononuclear cells; qPCR, quantitative polymerase chain reaction; TNF-α, tumor necrosis factor alpha; IL, interleukin; IFN-γ, interferon gamma; FOXP3, forkhead box P3; EBI3, Epstein–Barr virus–induced gene 3; TGF-β, transforming growth factor beta.*





**Supplementary Fig.4. *Differential expression of immune-related genes in blood from CTL*** ***(n = 17) and CNTL (n = 14) patients****.* Gene expression levels were quantified by qPCR in lymph node mononuclear cells isolated from CTL and CNTL patients. Red dots represent CTL patients and blue dots represent CNTL individuals. Data are presented as individual values with median ± interquartile range (IQR). The biomarkers include Granzyme B, TNF-α, IL-1β, IFN-γ, FOXP3, IL-12p35, EBI3, IL-17, and TGF-β. Statistical comparisons were performed using unpaired two-tailed t-tests. Non-significant comparisons are labeled as *ns*. A p < 0.05 was considered statistically significant.

*CTL, cervical tuberculous lymphadenitis; CNTL, non-tuberculous cervical lymphadenopathy; qPCR, quantitative polymerase chain reaction; ETA, endothelin A receptor; TNF-α, tumor necrosis factor alpha; IL, interleukin; IFN-γ, interferon gamma; FOXP3, forkhead box P3; EBI3, Epstein–Barr virus–induced gene 3; TGF-β, transforming growth factor beta.*


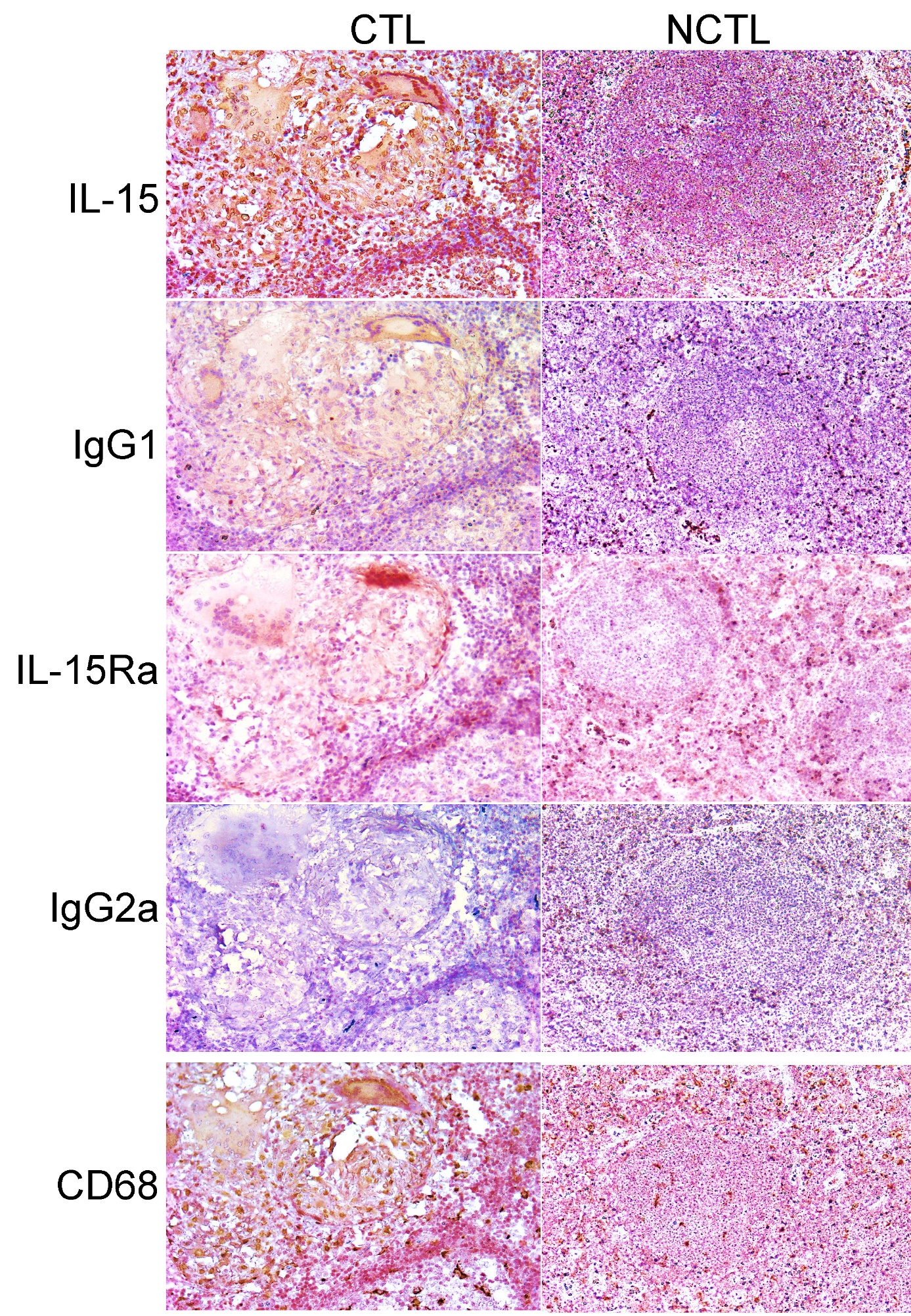


**A**


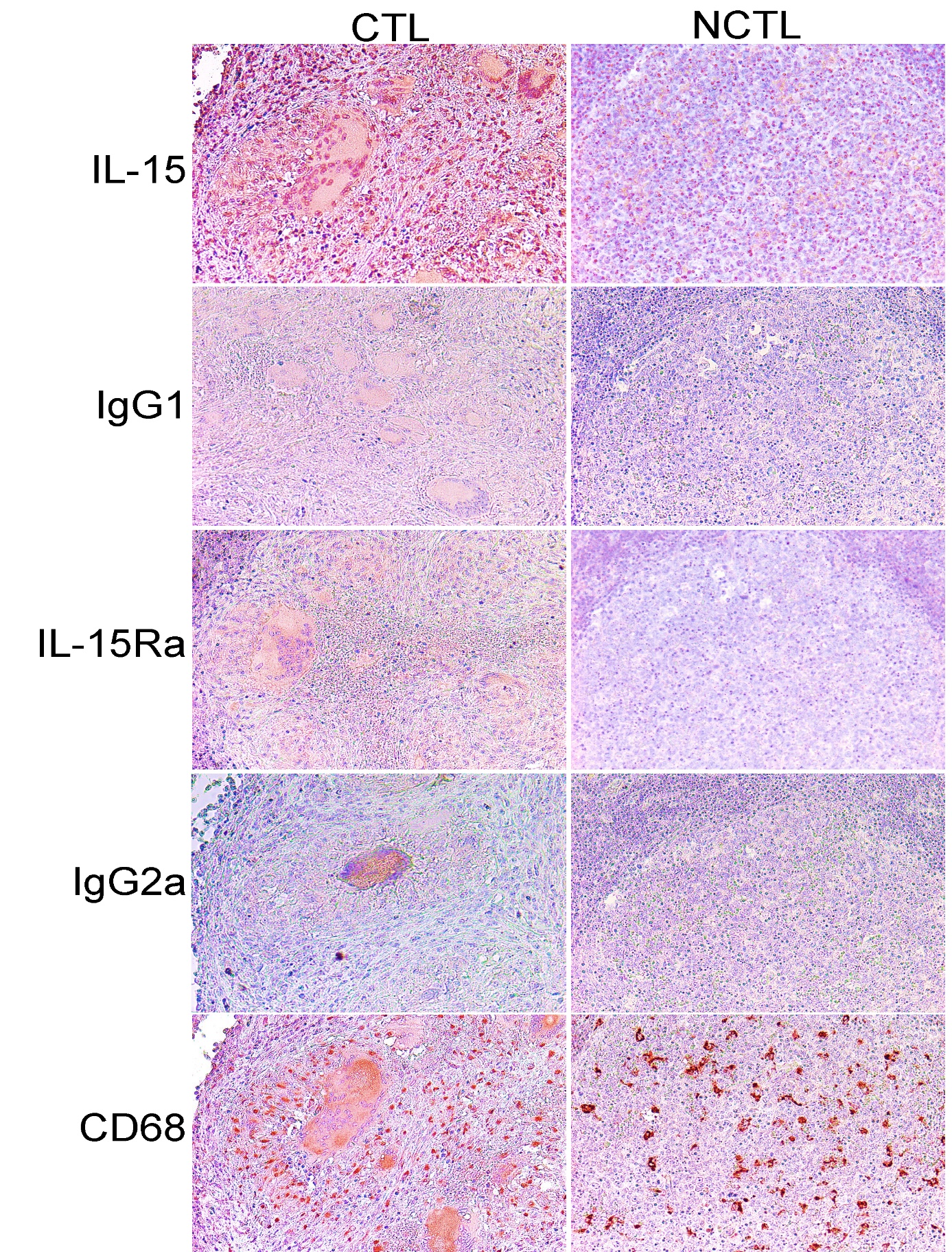


**B**

**Supplementary Fig.5:** Representative immunohistochemical staining of cervical lymph node sections from two different patients (A and B) with CTL and CNTL showing IL-15, IL-15Rα, and CD68 expression. IgG1 and IgG2a were used as isotype controls.
